# Realist Review of Care Models That Include Primary Care for Adult Childhood Cancer Survivors

**DOI:** 10.1093/jncics/pkac012

**Published:** 2022-02-16

**Authors:** Claire Snyder, Youngjee Choi, Katherine C Smith, Renee F Wilson, Christina T Yuan, Paul C Nathan, Allen Zhang, Karen A Robinson

**Affiliations:** 1 Department of Medicine, Division of General Internal Medicine, Johns Hopkins University School of Medicine, Baltimore, MD, USA; 2 Department of Health Policy and Management, Johns Hopkins Bloomberg School of Public Health, Baltimore, MD, USA; 3 Johns Hopkins Sidney Kimmel Comprehensive Cancer Center, Baltimore, MD, USA; 4 Department of Health, Behavior and Society, Johns Hopkins Bloomberg School of Public Health, Baltimore, MD, USA; 5 Division of Hematology/Oncology, The Hospital for Sick Children, Toronto, ON, Canada; 6 Departments of Pediatrics and Health Policy, Management & Evaluation, University of Toronto, Toronto, ON, Canada; 7 Department of Epidemiology, Johns Hopkins Bloomberg School of Public Health, Baltimore, MD, USA

## Abstract

Appropriate models of survivorship care for the growing number of adult survivors of childhood cancer are unclear. We conducted a realist review to describe how models of care that include primary care and relevant resources (eg, tools, training) could be effective for adult survivors of childhood cancer. We first developed an initial program theory based on qualitative literature (studies, commentaries, opinion pieces) and stakeholder consultations. We then reviewed quantitative evidence and consulted stakeholders to refine the program theory and develop and refine context-mechanism-outcome hypotheses regarding how models of care that include primary care could be effective for adult survivors of childhood cancer. Effectiveness for both resources and models is defined by survivors living longer and feeling better through high-value care. Intermediate measures of effectiveness evaluate the extent to which survivors and providers understand the survivor’s history, risks, symptoms and problems, health-care needs, and available resources. Thus, the models of care and resources are intended to provide information to survivors and/or primary care providers to enable them to obtain/deliver appropriate care. The variables from our program theory found most consistently in the literature include oncology vs primary care specialty, survivor and provider knowledge, provider comfort treating childhood cancer survivors, communication and coordination between and among providers and survivors, and delivery/receipt of prevention and surveillance of late effects. In turn, these variables were prominent in our context-mechanism-outcome hypotheses. The findings from this realist review can inform future research to improve childhood cancer survivorship care and outcomes.

Due to improved therapies and risk stratification, there are an estimated 400 000 childhood cancer survivors in the United States (1). Many childhood cancer survivors experience lifelong, chronic morbidities (eg, cardiomyopathy, subsequent malignancies) (2-6). However, risks differ based on cancer type and location, therapy, genetic predispositions, lifestyle behaviors, and comorbid conditions (7). Thus, the National Academy of Medicine recommends lifelong follow-up based on these factors (8).

Models of survivorship care can include specialized survivorship expertise, general oncology care, and/or primary care; the appropriate model for a given survivor is unclear. Barriers preclude childhood cancer survivors from receiving specialized long-term follow-up care (eg, distance to clinic) and from receiving quality survivorship care in primary care (eg, providers’ perceived discomfort treating survivors) (9,10). The National Cancer Institute (NCI) requested a realist review of models of survivorship care that include primary care to better understand the state of the science and, ultimately, improve childhood cancer survivorship care and outcomes through future research investments.

## Methods

### Review Purpose and Scope

This realist review addresses models of care that include primary care and resources for adult survivors of childhood cancer. A realist review addresses “What works, how, why, for whom, to what extent and in what circumstances, in what respect and over what duration?” (11) A realist review first describes the underlying ideas and assumptions about how an intervention is meant to work through an initial program theory, which is then refined through further literature review. Realist reviews also describe context-mechanism-outcome (CMO) hypotheses around this program theory. CMO hypotheses describe how contextual features (C) can influence mechanisms (M) through which an intervention or strategy is purported to produce outcomes (O).

Unlike systematic reviews, realist reviews do not summarize existing evidence of whether an intervention works. Rather, they explore theories regarding how interventions are intended to work and how different contexts affect the mechanisms by which they work to achieve the intended outcomes. Whereas systematic reviews have strict inclusion and exclusion criteria on studies to review and synthesize, realist reviews may draw from both directly and tangentially related literature. As such, literature searching in realist reviews is iterative to develop, and then refine, the program theory and explore emerging ideas. The outputs of systematic reviews are evidence syntheses; the outputs of realist reviews are theories and CMO hypotheses.

The NCI-specified aim of this realist review was to address how models of care that include primary care and related resources could be effective in providing survivorship care for adult survivors of childhood cancer—not whether they are effective. Similarly, the review did not compare effectiveness of models of survivorship care that do and do not include primary care. The review focused on the cancer-specific aspects of survivorship care, not treatment or the overall care of adult survivors of childhood cancer (eg, unrelated comorbid conditions, general preventive care). We included survivors of cancer diagnosed before age 21 years; for simplicity, we did not distinguish between childhood and adolescent survivors and refer to childhood cancer survivors throughout.

### Review Approach

In this realist review, we identified models of survivorship care; described available resources (eg, tools, training) for childhood cancer survivors, their families, and providers; developed an initial program theory about how these models of care and resources are intended to work; and developed CMO hypotheses to explain how different contexts shape the mechanisms through which the models of care and resources produce outcomes (Supplementary Figure 1, available online). Throughout the process, we engaged 8 stakeholders, including childhood cancer survivorship care clinical and research experts, patient advocates, and caregivers. A realist review expert provided methodologic guidance. Our protocol was posted for public comment, and detailed methods and results (eg, search strategies, data abstraction summaries) are available online (12). We followed the Realist and Meta-narrative Evidence Syntheses: Evolving Standards guidelines in conducting and reporting this review (13).

### Identify Models of Care and Resources

To identify models of care and catalog available resources, we consulted stakeholders and reviewed the literature.

### Develop Initial Program Theory

We developed an initial program theory by engaging stakeholders, exploring relevant theories, and reviewing conceptual articles regarding how models of care and resources are intended to work, for whom, and in what contexts (14,15). We examined existing theories related to access to care, knowledge specialization, care coordination, and uptake of resources. Based on the team’s expertise, these theories were most relevant to the assigned topic; had other relevant theories emerged, they also would have been considered. Consistent with this first stage in the realist review process, we reviewed opinion pieces, editorials, commentaries, and qualitative and mixed-methods studies about how models of care and resources are intended to work. We obtained stakeholders’ input on whether the initial program theory reflected their understanding of the issues and regarding additional theories that may be useful for integration (16).

### Refine Initial Program Theory

#### Literature Searches

To refine our program theory, we conducted an additional search of the literature, this time for quantitative evidence to support or refute our initial program theory (in contrast to the “conceptual” articles reviewed to develop the initial program theory). We searched PubMed, CINAHL, and PsycInfo using a search strategy informed by our initial program theory and not limited by study design or location. We also posted a federal register notice (17), searched Google Scholar, and used snowballing (searching references of studies) and berry-picking (finding information bit-by-bit using a range of sources including expert input) to identify additional studies (16,18,19). We screened abstracts and full-text articles using methods described by Carrieri (20). Each abstract was screened by 1 team member, with a random sample checked by another team member for consistency.

We included studies that provided information about models of care or resources for adult survivors of childhood cancer or cancer survivors of any age group. We excluded studies of individuals who were not disease free and studies on the transition from pediatric to adult care.

#### Data Extraction and Appraisal

One team member extracted evidence from each included article, including study design, purpose, population, and relevant details regarding models and resources. We identified findings related to the initial program theory and drew from study authors’ interpretations to inform possible CMO associations. Specifically, guided by the initial program theory, the team abstracted how the study findings or author discussions could contribute to our understanding of how different mechanisms in various contexts led to particular outcomes.

Although we intended to classify studies as making major, medium, or minor contributions to informing the program theory, so few studies qualified as “major” that, in practice, we prioritized literature focused on childhood cancer survivors (during childhood, adolescence, or adulthood) and supplemented with relevant evidence from adult survivors of adult cancers (20). In interpreting the findings, we considered their relevance to the initial program theory and their methodological rigor, using criteria adapted from Critical Appraisal Skills Programme (21).

#### Analysis and Synthesis

We reviewed data extractions during team meetings. We organized the studies by focus (eg, models of care, survivorship care plans [SCPs]), study design (eg, randomized controlled trials, prospective studies), and perspective (eg, survivor, provider), listing studies related to childhood cancer survivors first, followed by studies from other populations. We then organized the findings across studies by topic (eg, patient patterns of care, provider practices and preferences) using the childhood cancer survivorship studies supplemented with relevant findings from studies of adult survivors of adult cancers. Through this engagement with the study findings, the team 1) refined the program theory, 2) updated the list of program theory variables, and 3) developed the CMO hypotheses (20).

#### Refined Program Theory

We presented our refined program theory, updated variable list, and CMO hypotheses at a final stakeholder meeting to elicit feedback regarding whether our findings reflected their understanding and experience. Based on this feedback, we made final revisions to the refined program theory, variables, and CMO hypotheses.

#### Peer Review

The draft report was sent to peer reviewers and the stakeholders and simultaneously posted for 4 weeks for public comment.

## Results

Below, we describe our results from 1) the literature searches, 2) defining models of care, 3) identifying resources, 4) developing the initial program theory, 5) refining the program theory, and 6) developing CMO hypotheses (first focused on survivors, then focused on providers).

### Results of Searching

We reviewed 62 articles to develop our initial program theory and 135 articles for program theory refinement (Supplementary Figure 2, available online).

### Defining Models of Survivorship Care

There is no consistent taxonomy for survivorship models of care. For example, the American Society of Clinical Oncology describes 8 models of care, classified by provider(s) and setting: oncology specialist care, multidisciplinary survivorship clinics, disease- or treatment-specific survivorship clinics, general survivorship clinics, consultative survivorship clinics, integrated survivorship clinics, community generalist model, and shared care (22). However, the American Society of Clinical Oncology models are rarely formally evaluated in research. What is seen in the literature (and experienced by survivors) more often are patterns of care that occur not by design but due to circumstance.

Because research studies generally have limited data on patterns of care for classification of models, they tend to use broader categories. For example, Nathan et al. (7) organized care receipt as no health care, general medical care, general survivor-focused care, and risk-based survivor-focused care. Oeffinger et al. (23) focused on health-care interactions: general contact, general physical examination, cancer-related medical visit, and cancer center visit. Mueller et al. (24) organized care by provider type seen: primary care provider (PCP), specialty care physician, nurse practitioner or physician’s assistant, and survivorship clinic team. Surveys of providers have asked about continued care in pediatric oncology, referral to primary care, shared-care with primary care, and follow-up through a specialized long-term follow-up program (25-27). One systematic review simply categorized models as general practitioner (GP)–led vs shared care between a GP and pediatric oncology or a late-effects clinic (28).

Using the information from the literature and stakeholders, we defined 4 dimensions describing models of survivorship care that include primary care: 1) presence of particular expertise in survivorship (regardless of provider type or specialty), 2) PCP’s role in delivering survivorship care, 3) access to academic or cancer center support for survivors and/or providers, and 4) one-time or occasional consults vs longitudinal management (Box 1).

Box 1. Dimensions based on the literature and stakeholders describing models of survivorship care that include primary care and types of resources for childhood cancer survivors, their families, and providers^a^

Dimensions Defining Models of Care that Include Primary Care.

- Particular expertise in survivorship?
  - Is the survivorship expert trained in oncology, primary care, other?
  - Is the survivorship expertise MD, NP/PA, multidisciplinary?
- Role of PCP (main provider of survivorship care, provides survivorship care under the guidance of survivorship expert, provides primary care with no particular attention to survivorship).
- Access to academic or cancer center support for survivors and/or providers.
- One-time or occasional consults vs longitudinal management or unclear.

Types of Resources for Childhood Cancer Survivors, their Families, and Providers.

- Long-term follow-up guidelines.
- Educational materials directed at either survivor or family or care providers, regardless of media (ie, electronic, hard copy).
- In-person or virtual trainings (ie, workshops, conferences, continuing medical education courses) directed at either survivor or family or care providers.
- Survivor care documents (ie, survivor-specific standardized letters, treatment summaries, survivorship care plans).
- Survivorship care management processes (ie, expedited routes of contact for consultation, rereferral, support services; methods for digitizing and securely distributing health records; and other provider-to-provider and provider-to-survivor communications).
- Survivor supportive tools and services (in-person or digital), such as text messaging or peer navigator programs, support groups (in-person, telephone-based, or online), and professional psychosocial counselors (in-person, telephone-based, or online).

^a^MD = medical doctor; NP = nurse practitioner; PA = physician’s assistant; PCP = primary care provider.

### Identifying Resources

Regarding survivorship resources, surveys have asked about survivor-specific standardized letters; follow-up guidelines; expedited routes of contact; and websites, medical education, and pamphlets (26,27). A systematic review identified a well-organized transition, treatment summary, SCP, generalist provider education, and guidelines as components of successful follow-up (28). For this realist review, the term “resources” included guidelines, educational materials, trainings, documents, processes, and supportive tools and services (Box 1).

We identified 40 resources (Supplementary Table 1, available online): 23 survivor specific, 12 provider or researcher specific, and 5 directed at both. We identified 9 US and 6 international guidelines.

### Developing Initial Program Theory

Based on the stakeholders’ input and the literature, we developed an initial program theory and variable list (Supplementary Table 2, available online). The fractured US health-care system and availability of financial and other resources were identified as key variables at the system, provider, and survivor levels. In addition, coordination of care—among primary care and specialty providers and between providers and survivors—influences the care that is delivered and received and whether and when transition to primary care occurs. Survivor-specific characteristics (eg, developmental age, time since diagnosis, current treatment effects) are also important.

The stakeholders identified mortality, morbidity, quality of life, and costs as key final outcomes, summarized as “survivors live longer and feel better through high-value care.” High-value care is defined as “the best care for the patient, with the optimal result for the circumstances, delivered at the right price” (29). Intermediate outcomes relate to survivor knowledge and follow-up, summarized as “survivors feel confident about sharing their history, know their risks, recognize symptoms and problems, understand the care they need, are aware of the resources available to help them, and can access relevant care and services.” Resource effectiveness is determined by whether they are accessible, user-friendly, known, and trusted.

The Andersen Behavioral Model of Health Services Use (30) was the existing theory that best fit the identified factors from the initial stakeholder discussions. This model describes how environmental factors (health-care system, external environment) relate to population characteristics (predisposing characteristics, enabling resources, need) that influence health behavior (personal health practices, use of health services) that lead to outcomes (perceived health status, evaluated health status, consumer satisfaction). With minor modifications, the key factors identified by the stakeholders fit into the Andersen model categories. The stakeholders broadly supported the initial program theory’s description of the influence of models of care and resources for childhood cancer survivors. They suggested minor additions and refinements to the variable list.

### Refining Initial Program Theory

**Figure 1.**
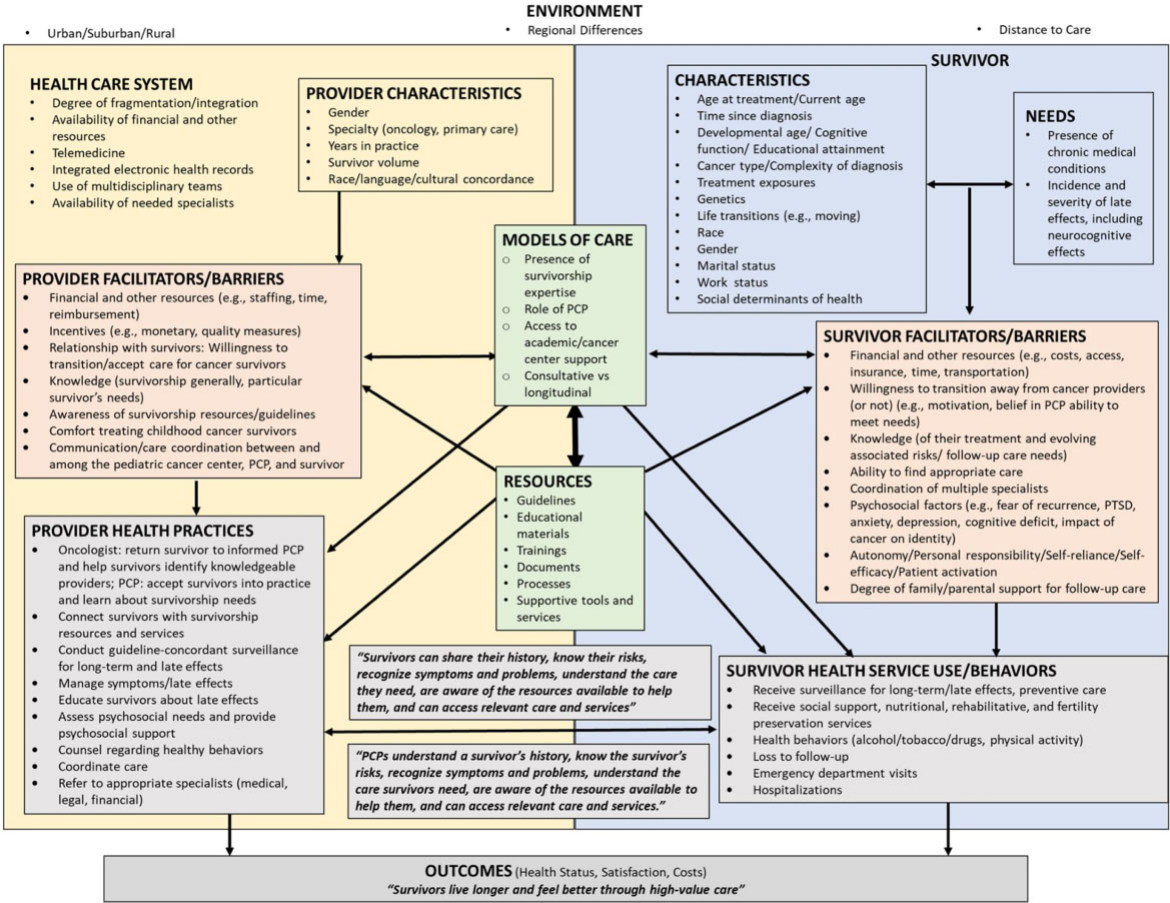
Refined program theory. Health system or provider factors (left) and survivor factors (right) exist in the background of the overall environment. The models of care and resources sit at the intersection of health system or provider factors and survivor factors. The health-care system attributes and provider factors are at the top left, and the survivor characteristics and needs are at the top right. The modifiable facilitators and barriers on the health system or provider side and survivor side are shown in the middle, on each side respectively. Intermediate outcome and process measures (provider health practices; survivor health service use or behaviors) are displayed near the bottom, with the final outcomes displayed at the very bottom. PCP = primary care provider; PTSD = posttraumatic stress disorder

Our review of the quantitative literature to refine the program theory provided at least some support for almost all variables in our initial list (Figure 1). The variables most commonly found in the literature included oncology vs primary care specialty; cancer type and complexity of diagnosis; survivor age, race, and gender; survivor and provider financial and other resources; survivor and provider knowledge; provider comfort with treating childhood cancer survivors; communication and coordination between and among providers and survivors; delivery and receipt of prevention and surveillance of long-term and late effects; and quality of life or health status and satisfaction. Variables seen less frequently include crisis events, genetics, emergency department visits, hospitalizations, and costs. The literature also identified some factors that we added to our variable list (shown in italics in Supplementary Table 2, available online). The most important change to the initial program theory was the addition of a PCP-focused intermediate outcome: “PCPs understand a survivor’s history, know the survivor’s risks, recognize symptoms and problems, understand the care survivors need, are aware of the resources available to help them, and can access relevant care and services.” In the final round of consultations, the stakeholders reported that our refined theory was consistent with their understanding based on their experiences with and expertise in childhood cancer survivorship care.

### Developing CMO Hypotheses

Guided by our program theory and the quantitative literature abstractions, we developed 7 CMO hypotheses. Consistent with our assigned topic and realist review methodology, the CMOs do not address whether models of care that include primary care are effective but, rather, in what contexts and via what mechanisms they could be effective.

These CMO hypotheses were primarily developed based on studies describing childhood cancer survivors (during childhood, adolescence, or adulthood), supplemented with evidence regarding adult survivors of adult cancers, where informative. For each CMO, we provide example evidence that supports, and in some cases refutes, the hypothesis. As with all realist reviews, the evidence provided is illustrative and not intended to be comprehensive.

### CMOs Focused on Survivor Intermediate Outcome

Table 1 summarizes the CMO hypotheses associated with the survivor-focused intermediate outcome.

**Table 1.** Context-mechanism-outcome hypotheses regarding how models of care that include primary care could be effective, focusing on intermediate outcomes for the survivor^a^

In the context of	This mechanism produces	Outcome (intermediate)	Outcome (final)
A) The availability of survivorship care plans, guidelines, and other resources	A1) Improved survivor knowledge A2) Information available to share with PCP to inform delivery of survivorship-related care	Survivors can share their history, know their risks, recognize symptoms and problems, understand the care they need, are aware of the resources available to help them, and can access relevant care and services.	Survivors live longer and feel better through high-value care.
B) Healthier survivors (perceived or actual)	B) Less perceived/actual need for survivorship-related care		
C) Survivors engaged in health-care system	C) Improved knowledge		
D) Survivor confidence in PCPs	D) Willingness to transition care		

a PCP = primary care provider.

#### Context A: SCPs, Guidelines, and Other Resources

The availability of SCPs, guidelines, and other resources is hypothesized to lead to the survivor-focused intermediate outcome via 1) survivors’ improved knowledge regarding their care needs, and 2) survivors’ sharing information with their PCPs. We also identified 4 subthemes: 1) benefits and harms of information, 2) importance of baseline knowledge, 3) resource “dose,” and 4) gender differences.

Regarding the first mechanism, multiple studies in childhood cancer survivors have shown that resources such as SCPs and/or follow-up care instructions are associated with improved knowledge, fewer unmet information needs, and better adherence to surveillance (31–33). There was also evidence that survivors valued the information from SCPs and other resources to share with their other providers. In a study of 111 adult survivors of pediatric and young adult cancer, of 30 respondents with an outside provider visit since SCP receipt, 33% gave the provider a copy of the form and 44% gave a copy to someone in their personal circle (34).

However, intentions to share the SCP do not always translate into action. In a cohort study of 20 families of a child with acute lymphoblastic leukemia, 96% of parents reported intending to share the SCP with someone, but only 61% had done so at the third follow-up, and only 35% of those with a PCP had shared it with them (35). Another study found that nearly one-half of childhood cancer survivors who had seen a physician since SCP receipt had shared it; most who had not seen a physician planned to share the SCP when they did (36). Further, the investigators found that PCPs ordered tests when the survivor presented them with information on risks. In a study of 5661 adult survivors of childhood cancer, Steele et al. (37) found that discussing cancer-related risks with a doctor is the strongest predictor of getting screened for late effects and that the physician’s access to the survivor’s cancer treatment summary statistically significantly predicted screening for relevant health risks.

In summary, survivors value the information and may intend to share it but do not always do so, and sharing this information with providers effectively can lead to guideline-concordant care.

The first subtheme associated with Context A discusses how resources and the information they provide can be both beneficial and detrimental. One study of adult survivors of pediatric and young adult cancer found that 14% reported being concerned by the SCP generally, and 28% were concerned by potential late effects (34). A study not specifically in childhood cancer survivors found greater reports of pain among survivors who had received SCP-type documents (38), but another found fewer reports of late effects (39). Speculations regarding the finding related to pain include that heightening cancer survivors’ awareness led to greater reporting or that patients undergoing more extensive treatment were more likely to both experience pain and receive SCP-type documents. As for survivors receiving follow-up care instructions reporting fewer late effects, the authors speculate survivors became aware of symptoms sooner, leading them to obtain care earlier and resulting in them reporting fewer problems.

A Dutch randomized controlled trial of SCPs in gynecologic cancer survivors delved into the impact of information. In this study, endometrial cancer survivors in the SCP arm reported greater concern about their illness, more emotional effects, and more symptoms (40). Ovarian cancer survivors in the SCP arm reported less trust that the treatment would cure their disease (41). The authors noted that these negative outcomes are not necessarily bad. For example, the endometrial cancer survivors had more cancer-related contact with their PCPs, which the authors speculated relates to the survivors’ greater awareness of cancer-related symptoms and the possibility that the SCPs empowered them to obtain necessary support (40). They also suggested that health-care providers may be reluctant to share information about potential late effects to avoid such negative consequences but that avoidance of the issue may limit survivors’ awareness and empowerment. For the ovarian cancer survivors, the authors noted that the decreased belief in the potential for cure gleaned from the SCP may be more realistic but that it is an issue that providers may be reluctant to address (41). A follow-up analysis examined differential effects of SCPs among “monitors,” who desire information about their disease, and “blunters,” who avoid information (42). SCPs were more beneficial to monitors, particularly those who did not have easy access to other information sources, such as the internet. Blunters in the SCP arm reported a greater impact of the disease on life and more concerns about the illness vs blunters in the control arm.

These studies raise 2 key questions: 1) Do harms reflect unnecessary concern or a more realistic understanding of the cancer’s implications? 2) To what extent should information delivery be tailored to the survivor’s preference?

The second subtheme associated with Context A identifies the importance of baseline knowledge. Several studies have found that resources improved knowledge more in survivors who initially knew less. In 1 study of adult survivors of childhood cancer, new patients were more likely to report learning new information from the SCP vs return patients (34). A study testing a survivorship clinic visit intervention in 369 adult survivors of childhood cancer found that survivors with the lowest baseline knowledge of therapy and therapy-related health risk had the greatest gains (43). Articles with similar findings in adult survivors of adult cancers have posited that resources’ failures to demonstrate impacts may be partially due to high levels of knowledge and/or few or no needs in the populations being studied (44,45). These findings raise the question of whether resources should be targeted to survivors with information deficits or needs.

The third subtheme associated with Context A relates to resource “dose.” To be effective, resources must deliver a sufficient “dose.” Several studies attributed the failure to find an effect of the studied resource to an insufficient dose (37,46,47). A brief, broad-based risk counseling intervention did not achieve a substantial long-term change in knowledge, health perceptions, or health practices in childhood cancer survivors (46). Similarly, the “relatively weak intervention dose” of a targeted (not tailored) newsletter page did not lead to increased medical follow-up in at-risk pediatric cancer survivors (37). Although tailored information is more consistently effective, effects are small (37). Evidence is needed to confirm whether more intensive interventions lead to greater resource effectiveness. However, as discussed below, practical considerations limit how much time and effort a resource can involve.

The fourth subtheme associated with Context A describes differences in gender  regarding engagement with and impact of resources. Female childhood cancer survivors had a small but statistically significantly greater improvement in knowledge following a multicomponent risk-counseling intervention vs males (46). Similarly, more women (72%) than men (59%) reported reading a newsletter in a study of childhood cancer survivors (37). In a single-arm study of high-risk childhood cancer survivors, more women than men visited the study website with survivorship resources (36). These results raise questions regarding whether resources should be tailored to different genders  based on how they engage with them.

#### Context B: Survivors Who Perceive Themselves to Be, or Are Actually, Healthier

Consistent with recommendations for risk-adjusted follow-up care (8), evidence suggests that models of care that include primary care could be effective for childhood cancer survivors who perceive themselves to be—or are actually—healthier (7,48–52). In a survey of 160 Swiss adolescent or young adult survivors, nonattenders of follow-up were more likely to rate models of care involving a GP or via telephone or questionnaire higher than attenders (50). Because nonattenders were also less likely to report late effects than attenders of follow-up, this finding supports the hypothesis that models of care that include primary care could be effective for survivors who are healthier. In another study, childhood cancer survivors who reported no morbidity on their baseline questionnaire were less likely to report receiving care on follow-up questionnaires, whereas survivors who reported any chronic health condition at baseline were more likely to report care at follow-up (48). However, not getting care is not equivalent to not needing care, and some survivors may not understand their risk for late effects. In 1 study, childhood cancer survivors reported not getting cardiomyopathy screening because they did not feel it was important (53). However, screening detected cardiomyopathy or other relevant abnormalities in more than 20% of those screened.

Thus, although survivors who are healthier may be more comfortable with follow-up in primary care, perceived health need is not equivalent to actual health, and survivors who do not understand their risks for late effects may not be receiving recommended screening and needed care.

#### Context C: Survivors Who Are Engaged in the Health-Care System

Being engaged in the health-care system (eg, physician visits) provides another context for survivors to gain the knowledge they need to receive appropriate care, though not universally (33,54,55). A Swedish study found that childhood cancer survivors who had no regular contact with health-care services were more likely to report that they had not received knowledge, treatment strategies, or guidance for coping with physical changes (54). Another study found that childhood cancer survivors who had a cancer-related checkup or visited a doctor more than 5 times in the past 2 years were more likely to receive surveillance for late effects (33). Male sex, lack of insurance, lower income, race (non-White or other), and less education are associated with childhood cancer survivors not being engaged in care (7,48,49,55).

These findings provide insights regarding the pathways to appropriate care receipt, with survivors engaged with the health-care system learning more about their care needs and receiving appropriate care.

#### Context D: Survivors Who Have Greater Confidence in Their PCPs

Although survivors who are confident in their PCPs might be more willing to transition their care, the evidence regarding this CMO hypothesis actually supports the converse: survivors are not confident in PCPs and prefer models of care that include cancer specialists (50,56). For example, Swiss adolescent or young adult survivors rated medical oncologist involvement most highly for survivorship care (50). Even though the respondents were surveyed at least 5 years after diagnosis, their biggest concerns were cancer relapse and late effects, and survivors may perceive medical oncologists as best suited to deal with these issues. However, in a Dutch survey of childhood cancer survivors, 88% were satisfied with the care given by local family doctors, and only 14% thought their local family doctor’s knowledge of their medical history was inadequate (57).

The limited evidence regarding this CMO hypothesis suggests that work is required to increase cancer survivors’ confidence in PCPs to facilitate models of care that include primary care if evidence supports PCPs’ effectiveness in delivering survivorship care.

### CMOs Focused on Provider Intermediate Outcome

Table 2 summarizes the CMO hypotheses associated with the provider-focused intermediate outcome.

**Table 2.** Context-mechanism-outcome hypotheses regarding how models of care that include primary care could be effective, focusing on intermediate outcomes for the primary care provider^a^

In the context of	This mechanism produces	Outcome (intermediate)	Outcome (final)
A) The availability of survivorship care plans, guidelines, and other resources	A) Information available to guide the PCP in delivering survivorship-related care	PCPs understand a survivor’s history, know the survivor’s risks, recognize symptoms and problems, understand the care survivors need, are aware of the resources available to help them, and can access relevant care and services.	Survivors live longer and feel better through high-value care.
B) PCP shared care with oncologist	B) Support from the oncologist to aid the PCP in delivering survivorship-related care		
C) More experience caring for childhood cancer survivors	C) Greater comfort caring for childhood cancer survivors		

a PCP = primary care provider.

#### Context A: SCPs, Guidelines, and Other Resources

PCPs value follow-up guidelines, SCPs, and similar documents for supporting their delivery of survivorship care to childhood cancer survivors (26,27,58–61). There is also evidence that PCPs use these resources and that the resources promote quality survivorship care. Among PCPs caring for childhood cancer survivors who recalled receiving an SCP as part of a research study, 75% reported often or always reviewing the plan, and 42% reported discussing the SCP with the survivor (61). PCP possession of an SCP has also been associated with increased adherence to recommended surveillance (33).

However, evidence exists that these resources currently have limited reach and effectiveness (58,61). Surveys of PCPs found that a minority recall receiving a cancer treatment summary or SCP, being aware of the Children’s Oncology Group (COG) late effects guidelines, or feeling comfortable recognizing late effects or providing other aspects of survivorship care (58,61). The limitations of passive SCP distribution suggest the need for better ways to deliver information to PCPs (61). However, another study found substantial improvements in adherence to the COG guidelines between 2003 and 2016 and speculated that physician awareness of COG guidelines may be growing (33).

In summary, the potential value of these resources in promoting effective childhood cancer survivorship care has yet to be fully realized. We identified 3 related subthemes: 1) lack of awareness, 2) managing information in electronic health records (EHR), and 3) resource “dose.”

The first subtheme associated with Context A addresses lack of awareness. In a study of childhood cancer survivorship care, only 51% of PCPs sent an SCP as part of the study recalled receiving it (61). PCPs in a survey regarding primary care for adult breast cancer survivors reported being unaware of the SCP (73%), difficulty locating it (30%), and finding needed information faster via another mechanism (15%) (62). These barriers were reported even though SCPs are standardly housed in the EHR problem list within their institution. The investigators speculated that their institution may have yet to reach a “critical mass” of SCP provision to facilitate PCPs’ awareness of and ability to use them. They suggested the need for “primary care-centered design of SCP format and content, location in the EHR, and the ability to ‘push’ relevant or needed survivorship information to primary care at the right time.” In another study, only 34% of providers for adult survivors of adult cancers recalled receiving the SCP or could locate it (63). The authors of this study compared the SCP with “a needle in a haystack” for health-care professionals across institutions who use different EHRs or paper charts.

Knowing that an SCP exists is a critical first step to being able to use it. EHRs can both help and hinder that process.

The second subtheme associated with Context A discusses managing information in EHRs. Multiple studies in adult survivors of adult cancers addressed the role of the EHRs (40,41,64,65). Advantages of using EHRs to create and provide SCPs include reducing time and resources to compile information, producing an electronically searchable document, and facilitating plan updates when needed (65). However, for EHRs to support effective SCP use, discrete data capture is required, organization policies and technologies should support clinician needs, and survivorship-related tasks must be clearly assigned (65). Perhaps because of these barriers, surveys of cancer programs and cancer care providers found that EHR systems used to create SCPs were lacking and/or underused (66,67). EHRs’ potential roles to create, deliver, and manage SCPs require further development.

The third subtheme associated with Context A relates to resource “dose.” Similar to survivors, there is a tension between resources delivering a sufficient “dose” to providers and demands on PCPs’ time. In 1 study, no participating PCP visited a virtual information center for childhood cancer survivors and their providers (36). In another example, only 24 of 46 eligible PCP–survivor dyads enrolled in a study of a telemedicine transition visit, partially due to PCPs’ hesitation with using the study-provided telemedicine equipment (68). One stakeholder suggested appropriate reimbursement would encourage providers to undertake these activities.

Similar to survivors, providing a sufficient “dose” of information needs to be balanced against the effort providers can invest.

#### Context B: Shared Care With Oncologist

Like survivors, PCPs prefer models of care that include cancer specialists and shared care (26,27,60,61). In surveys regarding childhood cancer survivorship, PCPs prefer working in collaboration with a cancer center–based physician or long-term follow-up clinic (26,27). Communication and collaboration between pediatric oncologists and PCPs, and documents such as SCPs, are important to support shared care (50,53,57,60). However, in a Swiss survey, 94% of GPs reported wanting more support from oncologists regarding follow-up for childhood cancer survivors (59). Two studies in adult survivors of adult cancer similarly support the importance of communication and connections between providers (69,70).

When implemented effectively, shared-care models provide close connections and quality communication so that PCPs have the information they need from cancer specialists to deliver appropriate survivorship care.

#### Context C: More Experience Caring for Childhood Cancer Survivors

Limited evidence suggests that PCPs with more experience caring for cancer survivors are more comfortable doing so and better adhere to guidelines (26,27). However, most PCPs treat few if any childhood cancer survivors (26,27,58,61). In 2 surveys, only 51%-58% of PCPs reported caring for 1 or more childhood cancer survivors in the past 5 years (26,27). On average, PCPs reported caring for 1 childhood cancer survivor in the past 5 years; 84% reported caring for only 1 (61). Only 34% had seen at least 5 late effects and 45% had seen a late effect of grade 3 or higher (61). These themes were echoed in a study of 86 PCPs regarding care for adult survivors of hematologic malignancies and hematopoietic cell transplantation (71). PCPs who had cared for more survivors felt more confident and perceived fewer barriers to doing so. They also were more likely to report having discussed screening and late effects with patients.

More research supporting the connections among PCP experience, comfort, and effectiveness caring for childhood cancer survivors is needed. However, even if these relationships are supported, operationalizing an approach to concentrate childhood cancer survivors in a PCP’s practice would be challenging.

## Discussion

### Summary of Findings

This realist review addresses how models of survivorship care that include primary care and resources can be effective for adult survivors of childhood cancer, their families, and providers. Effectiveness for both resources and models is defined by survivors living longer and feeling better through high-value care. Intermediate measures of effectiveness evaluate the extent to which survivors and providers understand survivors’ history, risks, symptoms and problems, health-care needs, and available resources. Thus, the models of care and resources are intended to provide information to survivors and/or PCPs to enable them to obtain or deliver appropriate care. The variables from our program theory that were seen most consistently in the literature include oncology vs primary care specialty, survivor and provider knowledge, provider comfort treating childhood cancer survivors, communication and coordination between and among providers and survivors, and delivery or receipt of prevention and surveillance of late effects. In turn, these variables were prominent in our CMO hypotheses.

Our discussion of the CMO hypotheses also describe why they may not be effective in achieving the desired outcomes. For example, we discuss the importance of information from resources, but we also discuss how information may be both beneficial and harmful, how it may be more effective for some populations (eg, survivors with lower baseline knowledge), and the challenges of delivering the appropriate information “dose.” Similarly, we note PCPs’ lack of awareness of resources, the possibilities and problems of information in the EHR, and, again, delivering a sufficient “dose” of information without requiring undue burden. Thus, our CMOs describe how various mechanisms could be effective—and why they may not be.

### Strengths and Limitations

The strengths and limitations of this realist review inform the interpretation of our findings. NCI funding may have unintentionally influenced study selection and reporting. Although the rigor and relevance of reviewed studies were noted in the data abstraction to inform interpretation of study findings, this approach used in realist reviews does not formally grade the evidence as in systematic reviews. Each study was abstracted by only 1 team member, which could raise concerns of bias. However, the program theory and CMO hypotheses are based on the themes and concepts that emerge from the literature (as with qualitative research) rather than single studies. To minimize bias, the program theory and CMO hypotheses were discussed both within the team and with our stakeholder experts to ensure consistency with their understanding of the field.

We were charged with addressing how models of survivorship care that include primary care could be effective—not whether they are effective. This review focused on the cancer-specific aspects of survivorship care, not the overall care of adult survivors of childhood cancer (eg, unrelated comorbid conditions, general preventive care) where the PCP’s role is clearer.

Although this realist review aimed to address “models of care,” the literature generally provides only evidence regarding “patterns of care” (ie, who was seen where and received what) rather than formal evaluations of care models. Thus, we faced the challenge of conducting a realist review of multiple ill-defined patterns of care, when ideally a realist review focuses on a “well-defined program” (72). Also, data regarding final outcomes, particularly mortality, are sparse (eg, are survivors who are more adherent to recommended surveillance more likely to live longer?).

Strengths of our review include the investigation of a wide range of articles, in multiple contexts, internationally. For initial program theory development, we focused on commentaries, editorials, and qualitative and mixed-methods articles that described intended operations and outcomes of models of care and resources. To refine the program theory, we focused on quantitative studies that could inform our program theory revisions and CMO hypothesis development. We included studies examining models of care and resources not only for adult survivors of childhood cancer but also for child or adolescent survivors as well as adult survivors of adult cancer. Although the data from other populations added insights informing our CMOs, the generalizability of these findings to adult survivors of childhood cancer requires further exploration. We did not restrict our literature review to studies in the United States, although the applicability of studies from countries with different health systems may be limited.

During the conduct of this review, the world experienced the transforming effects of the COVID-19 pandemic, as reflected in our program theory variable list as “crisis events.” However, the literature has not even begun to reflect how medical care in general, and cancer survivorship care in particular, may be changed. For example, where telemedicine was relatively rare in 2019, it became commonplace, and in some cases dominant, in 2020 (73).

As described by one of our stakeholders, the pandemic further emphasized 2 questions related to this review: 1) Who needs to be seen in specialty care and who can be followed-up in their own community? 2) For those followed-up in the community, how can the knowledge that survivors and PCPs need to receive or deliver quality care be effectively transferred? These questions represent the crux of the issues that require further research.

## Conclusion

In combination, the products of this realist review (ie, the program theory and CMO hypotheses) provide valuable insights into how, for whom, in what contexts, and via what mechanisms models of care that include primary care and resources could be effective for adult survivors of childhood cancer. The NCI and others can use this information to inform future research investment regarding 1) which survivors can be seen in specialized vs community settings and 2) how to ensure that survivors and PCPs have the information they need to receive or deliver quality survivorship care.

## Funding

This work was supported by the Agency for Healthcare Research and Quality (AHRQ) under Contract No. 75Q80120D00003/Task Order 75Q80121F32005 from the Agency for Healthcare Research and Quality (AHRQ), U.S. Department of Health and Human Services (HHS). The National Cancer Institute (NCI) of the National Institutes of Health (NIH) funded the report.

## Notes

**Role of the** **funder:** The funders had no role in the design, execution, analysis, or publication of this study.

**Disclosures:** Dr Snyder receives research funding from Genentech and Pfizer through Johns Hopkins, consults to Janssen via Health Outcomes Solutions, and previously received royalties from UptoDate for authorship related to survivorship. YC has received salary support from a Merck Foundation grant related to cancer survivorship. Dr Nathan received an honorarium through the contract supporting this project (AHRQ under Contract No. 75Q80120D00003/Task Order 75Q80121F32005). All other authors have no conflicts to declare.

**Author** **contributions:** Conceptualization (CS, KAR), Data Curation (CS, RFW, AZ), Formal analysis (CS), Funding acquisition (CS, KAR), Investigation (all authors), Methodology (CS, KAR, RFW), Project administration (CS, RFW, AZ, KAR), Resources (RFW, KAR), Supervision (CS, KAR, RFW), Validation (CS, KAR), Visualization (CS, RFW), Writing—original draft (CS), Writing—review and editing (all authors).

**Disclaimers:** The findings and conclusions in this document are those of the authors, who are responsible for its contents; the content does not necessarily represent the official views of or imply endorsement by AHRQ or the U.S. Department of Health and Human Services. AHRQ retains a license to display, reproduce, and distribute the data and the report from which this manuscript was derived under the terms of the agency’s contract with the author.

## Data Availability

The data underlying this are publicly available in the Systematic Review Data Repository (SRDR).

## Supplementary Material

pkac012_Supplementary_Data
