# Supplementary material for: Realist Review of Care Models That Include Primary Care for Adult Childhood Cancer Survivors
Source: JNCI Cancer Spectr. 2022 Feb 16;6(2):pkac012. doi: 10.1093/jncics/pkac012 (PMC8946685; doi:10.1093/jncics/pkac012)

Supplementary Material

Supplementary Table 1. List of survivorship resources available to cancer survivors, their families, and their medical care providers

Resource	Link	For Cancer Survivors	For Providers	Description
American Cancer Society: Survivorship: During and After Treatment	https://www.cancer.org/treatment/survivorship-during-and-after-treatment.html	Yes	No	Information and tips on staying active and healthy during and after cancer treatment for cancer survivors. There is also information on dealing with the possibility of cancer recurrence, and stories about other people whose lives have been touched by cancer.
American Cancer Society: Tools for Cancer Survivors and Caregivers	https://www.cancer.org/health-care-professionals/national-cancer-survivorship-resource-center/tools-for-cancer-survivors-and-caregivers.html	Yes	No	Guidance documents for cancer survivors, including “Life After Treatment Guide” and a “Life After Treatment Guide” for Native Americans and Alaska Natives.
American Cancer Society: Tools for Health Care Professionals	https://www.cancer.org/health-care-professionals/national-cancer-survivorship-resource-center/tools-for-health-care-professionals.html	No	Yes	Cancer survivorship care tools and resources for providers, organizations, and communities.
American Institute for Cancer Research	https://www.aicr.org/?gclid=CI6fkJ6Y1rECFYao4AodiE0Aqw	No	Yes	Monthly updates with resources, tools, and guidance for healthcare professionals
American Society of Clinical Oncology: Cancer.Net[a]	https://www.cancer.net/survivorship/survivorship-resources	Yes	No	Cancer.Net provides timely, comprehensive information to help patients and families make informed health care decisions.
American Society of Clinical Oncology: Survivorship Compendium	https://www.asco.org/practice-policy/cancer-care-initiatives/prevention-survivorship/survivorship/survivorship-compendium	No	Yes	A repository of tools and resources for oncology providers to implement or improve survivorship care within their practices. The compendium serves as an accompaniment to the educational opportunities and clinical-guidance ASCO offers on survivorship care.
Cancer + Careers	https://www.cancerandcareers.org/en	Yes	No	Provide resources for cancer patients and survivors on thriving in the workplace, providing expert advice, interactive tools, and educational events.

Cancer Care	https://www.cancercare.org/	Yes	No	Providing comprehensive services including case management, counseling, and support groups over the phone, online, and in-person, educational workshops, publications and financial and co-payment assistance.
Cancer Financial Assistance Coalition	https://www.cancerfac.org/	Yes	No	Coalition of organizations helping cancer patients find financial and practical help (e.g., home care, housing, fertility assistance, genetic testing).
Cancer Match	https://cancermatch.com/	Yes	No	A cancer survivor networking and dating website.
Cancer Support Community	https://www.cancersupportcommunity.org/	Yes	No	Non-profit network of support organizations, hospitals, and clinic partnerships delivering free support and navigation services for cancer patients and families.
Cancer Support Community: Gilda's Club	https://www.cancersupportcommunity.org/	Yes	No	A member of the Cancer Support Community, offers support through counseling, connecting patients with a wider patient and caregiver community, and professionally led programs.
Cancer SurvivorLink	https://www.cancersurvivorlink.org/	Yes	Yes	A patient-centric communication tool and mechanism for cancer survivors providing repository for key health documents and ability to share their online health records with providers.
Centers for Disease Control & Prevention: Cancer Survivors	https://www.cdc.gov/cancer/survivors/index.htm	Yes	Yes	CDC website with guidance and information for cancer patients and survivors, caregivers, and health care providers.
Children's Oncology Group†	http://survivorshipguidelines.org/	No	Yes	Children's Oncology Group guideline is a resource for healthcare professionals who provide ongoing care to survivors of pediatric malignancies.
Dana-Farber Cancer Institute: Continuing Medical Education	https://www.dana-farber.org/for-physicians/education-and-training/continuing-medical-education/	No	Yes	Offering continuing medical education courses, sharing advances in cancer research and treatment with the healthcare community.
George Washington University Cancer Center: Advancing Patient-Centered Cancer Survivorship Care Toolkit	https://smhs.gwu.edu/cancercontroltap/resources/advancing-patient-centered-cancer-survivorship-care-toolkit	No	Yes	A toolkit to support training and technical assistance from Comprehensive Cancer Control Programs/Coalitions to health care providers/organizations to improve

				patient-centered cancer survivorship care in their state, tribe or territory.
Job Accommodation Network	https://askjan.org/	Yes	No	Offers guidance on workplace accommodations and disability employment issues.
JourneyForward	https://www.journeyforward.org/	Yes	No	Health website offering information on important health related topics, including cancer symptom management and post-treatment care.
LIVESTRONG	https://www.livestrong.org/	Yes	No	Non-profit organization providing support for people affected by cancer. Website offers contact information and list of available programs for cancer patients and survivors.
LIVESTRONG Survivorship Centers of Excellence	https://www.livestrong.org/who-we-are/news/livestrong-survivorship-centers-excellence-network-celebrates-10-years-success	Yes	No	Website celebrates the LIVESTRONG Survivorship Centers of Excellence’s 10 year anniversary and summarizes the work done by the centers and foundation.
National Cancer Survivors Day Foundation	https://ncsd.org/	Yes	No	The nonprofit National Cancer Survivors Day Foundation provides free guidance, education, and networking to hospitals, support groups, and other cancer-related organizations that host NCSD events in their communities.
National Coalition for Cancer Survivorship	https://canceradvocacy.org/	Yes	No	A survivor-led cancer advocacy organization, advocating for research, policy, and providing tools for self-advocacy.
NIH/NCI Cancer Survivorship	https://www.cancer.gov/about-cancer/coping/survivorship	Yes	No	Offers information for cancer patients to help cope with the transition to post-treatment.
NIH/NCI: Cancer-Related Post-traumatic Stress (PDQ®)–Patient Version	https://www.cancer.gov/about-cancer/coping/survivorship/new-normal/ptsd-pdq	Yes	No	The National Cancer Institute’s Physician Data Query on post-traumatic stress targeted for patients, providing summaries on the latest published information on cancer prevention, detection, genetics, treatment, supportive care, and complementary and alternative medicine.
NIH/NCI: Office of Cancer Survivorship	https://cancercontrol.cancer.gov/ocs/resources	Yes	Yes	Offers resources and information for many different audiences, including survivors and caregivers, researchers,

				health care professionals, and advocates.
NIH/NCI: Office of Cancer Survivorship	https://cancercontrol.cancer.gov/ocs/resources/survivors/follow-up-medical-care	No	Yes	Links to resources for patients to assist in dialogue with physicians on follow-up care.
NIH/NCI: SEER-CAHPS	https://healthcaredelivery.cancer.gov/seer-cahps/	No	Yes[a]	A resource for quality of cancer care research based on a linkage between the NCI's Surveillance, Epidemiology and End Results (SEER) cancer registry data and the Centers for Medicare & Medicaid Services' (CMS) Medicare Consumer Assessment of Healthcare Providers and Systems (CAHPS®) patient surveys.
Northwestern University: Oncofertility	https://www.nm.org/healthbeat/medical-advances/oncofertility	Yes	No	Offers resources on oncofertility, such as options for fertility treatment and methods to preserve a child’s fertility before cancer treatment.
OncoLink: OncoLife Survivorship Care Plan	https://oncolife.oncolink.org/	Yes	No	A program providing cancer survivors with information regarding the health risks they face as a result of cancer therapies, and resources to develop a cancer survivorship care plan.
Passport for Care (with the Children’s Oncology Group)	https://cancersurvivor.passportforcare.org/en/	Yes	Yes	A free online resource tool for childhood cancer survivors allowing them to access their treatment summary and follow-up care recommendations.
Patient Advocate Foundation	https://www.patientadvocate.org/	Yes	No	A non-profit organization offering case management services and financial aid to Americans with chronic, life threatening, and debilitating illnesses.
Save My Fertility	https://www.savemyfertility.org/	Yes	No	An online fertility preservation toolkit for adult cancer patients to learn more on preserving fertility before and during cancer treatment.
St. Jude Children’s Research Hospital: Childhood Cancer Survivor Study Tools and Documents	https://ccss.stjude.org/tools-and-documents.html	No	Yes	Tools and documents for the St. Jude Children’s Research Hospital’s Childhood Cancer Survivor Study.
St. Jude Children’s Research Hospital: Cure4Kids	https://www.cure4kids.org/	No	Yes	An online resource for healthcare professionals to enhance the care for children with cancer.
St. Jude Children’s Research Hospital: Together	https://together.stjude.org/en-us/life-after-cancer.html	Yes	No	Offers resources for childhood cancer survivors on late effects, survivorship care plans, etc.

Stupid Cancer®‖	https://stupidcancer.org/get-help/resources/	Yes	No	Provides links to resources for adolescent and young adults with cancer to find resources and help on topics such as college and career, mental health, caregiving, and finances.
University of Pennsylvania: OncoLink	https://www.oncolink.org/	Yes	Yes	Provides resources and tools for oncology healthcare professionals, as well as information for patients on cancer, diagnosis, treatment, coping, and survivorship.
University of Texas: MD Anderson Cancer Center Continuing Medical Education	https://www.mdanderson.org/education-training/professional-education/cme-conference-management.html	No	Yes	Source of information on Continuing Professional Education/Conference Management conferences, e-learning activities, and external educational partners
UptoDate	https://www.uptodate.com/contents/table-of-contents/oncology/cancer-survivorship	No	Yes	A source of reference with links to most up to date evidence and clinical guidance on cancer survivorship related topics.
Individual guidelines/reports				
Institute of Medicine report: Childhood Cancer Survivorship: Improving Care and Quality of Life	Hewitt M, Weiner SL, Simone JV. Childhood Cancer Survivorship: Improving Care and Quality of Life. Washington (DC): National Academies Press (US); 2003	No	Yes	Guideline outlining a comprehensive policy agenda linking improved health care delivery, investments in education and training, and expanded research to improve the long-term outlook for survivors of childhood cancer.
Institute of Medicine report: Ensuring Quality Cancer Care	Hewitt M, Simone JV. Ensuring Quality Cancer Care. Washington DC. National Academy Press.1999	No	Yes	A status report on current health services (circa 1999) and issues related to medical coverage, social and economic status, patient beliefs, physician decision-making, and other factors.
Institute of Medicine report: From Cancer Patient to Cancer Survivor: Lost in Transition	Hewitt M, Greenfield S, Stovall E. From Cancer Patient to Cancer Survivor: Lost in Transition. Washington DC. National Academy Press.2006	No	Yes	A book focusing on survivors of adult cancer during follow-up phase post primary treatment.
President's Cancer Panel Annual Report 2003–2004, Living Beyond Cancer: Finding a New Balance	https://deainfo.nci.nih.gov/advisory/pcp/annualReports/pcp03-04rpt/Survivorship.pdf	No	Yes	Recommendations for cancer survivorship compiled from meetings of the President's Cancer Panel involving stakeholders such as survivors, caregivers, advocates, providers, insurers, Federal and State government employees, media representatives, and support organization representatives.
Shepherd et al. (2010)	Shepherd EJ, Woodgate RL. Cancer survivorship in children and young adults:	No	Yes	An article providing a conceptual framework helping to define survivorship

	a concept analysis. J Pediatr Oncol Nurs. 2010 Mar-Apr;27(2):109-18. Doi: 10.1177/1043454209349807. PMID: 20044589.			and useful for research and clinical practice in pediatric oncology.
NCCN: Guidelines for Patients	https://www.nccn.org/patients/guidelines/cancers.aspx	Yes		National Comprehensive Cancer Network's guidelines for cancer patients.
NCCN: Clinical Practice Guidelines	https://www.nccn.org/patients/clinical/default.aspx	No	Yes	National Comprehensive Cancer Network's guidelines for clinicians and health professionals on cancer treatments.
COG: Survivorship Guidelines	https://childrensoncologygroup.org/index.php/survivorshipguidelines	No	Yes	Cancer survivorship guidelines for clinicians who are providing ongoing healthcare to survivors.
ASCO: Patient and Survivor Care	https://www.asco.org/research-guidelines/quality-guidelines/guidelines/patient-and-survivor-care	No	Yes	Source of clinical guidelines on cancer treatment and survivor care.
International guidelines/reports				
Dutch Childhood Oncology Group	https://www.dcog-ectc.nl/	No	Yes[a]	The Dutch Childhood Oncology Group - Early Clinical Trial Consortium (DCOG-ECTC) implements clinical trials in children with cancer. The website provides tools for investigators in participating DCOG-ECTC studies.
International Guideline Harmonization Group	www.ighg.org	No	Yes	An organization that develops international guidelines for the long-term follow-up of childhood, adolescent, and young adult cancer survivors.
PanCareSurFup	https://www.pancaresurfup.eu/	No	Yes	An organization consisting of 16 European institutions establishing guidelines for follow-up; provide training and workshops to stakeholders.
Scottish Intercollegiate Guidelines Network	https://www.sign.ac.uk/our-guidelines/	No	Yes	Healthcare group to improve the quality of healthcare for patients in Scotland through development and dissemination of clinical guidelines.
Union for International Cancer Control: Sustainable Development Goals	https://www.uicc.org/search/site/?f%5B0%5D=sm_index_page_type%3AResources	No	Yes	Union for International Cancer Control (UICC) represents over 1200 organizations including major cancer societies, ministries of health and patient groups, providing an avenue for advocacy, guidelines, and policy development.

United Kingdom Children's Cancer and Leukaemia Group	https://www.cclg.org.uk/	Yes	Yes	A professional association providing advice and information to government agencies, and childhood cancer survivors on childhood cancer, as well as funding research.

[a]Resources for researchers. CAHPS = Medicare Consumer Assessment of Healthcare Providers and Systems; NCI = National Cancer Institute; NIH = National Institutes of Health; SEER = Surveillance Epidemiology and End Results data

Supplementary Table 2. Variables from the Initial Program Theory and Refined Program Theory[a]

Category	Variables
Environment	Urban, suburban, rural<br>Distance to clinic<br>*Regional differences*<br>Crisis events
Health care system	Degree of fragmentation/integration<br>Availability of financial and other resources<br>Telemedicine<br>Integrated electronic health records<br>Use of multidisciplinary teams<br>Availability of needed specialists
Provider characteristics	Identity/Specialty (oncology, primary care)<br>Years in practice<br>Race/language/cultural concordance<br>*Gender*<br>*Survivor volume*
Survivor characteristics	Age at treatment<br>Time since diagnosis *(longitudinal and calendar time)*<br>Developmental age/Cognitive function/Educational attainment<br>*Cancer type/*complexity of diagnosis<br>Treatment exposures<br>Genetics<br>Life transitions (e.g., marriage, moving)<br>Race<br>Gender<br>~~*Education*~~<br>*Marital status*<br>Social determinants of health<br>*Work status*<br>*Current age*
Survivor needs	Presence of chronic medical conditions<br>Incidence and severity of late effects, including neurocognitive effects
Facilitators/barriers	

Provider	Financial and other resources<br>Clinic staffing<br>Time<br>Reimbursement<br>Volume<br>Incentives (e.g., monetary, quality measures)<br>Relationship with survivors<br>Willingness to transition away (oncology)/accept (PCP) care for cancer survivors<br>Knowledge<br>About childhood cancer survivors' needs generally<br>About a particular survivor's needs (e.g., through a survivorship care plan, access to medical records)<br>Awareness of survivorship resources/guidelines<br>Comfort treating childhood cancer survivors (relates to knowledge base and professional expertise)<br>Communication and care coordination between and among the pediatric cancer center, the primary care provider, and the survivor
Survivor	Financial and other resources<br>Financial costs (e.g., reimbursement, ability to pay co-pay)<br>Access to care<br>Health insurance<br>Time<br>Transportation<br>Willingness to transition away from cancer providers (or not)<br>Motivation<br>Provider influences<br>Belief in PCP ability to meet their needs<br>Knowledge<br>Of their treatment and evolving associated risks<br>Of their evolving follow-up care needs<br>Ability to find appropriate care<br>Coordination of multiple specialists<br>Psychosocial factors<br>Fear of recurrence<br>Post-traumatic stress disorder<br>Anxiety<br>Depression<br>Cognitive deficit<br>Impact of cancer on identity<br>Autonomy/Personal responsibility/Self-reliance/Self-efficacy/Patient activation<br>Degree of family/parental support for follow-up care<br>*Awareness and availability of (culturally appropriate) resources*

Provider health practices	*Oncologist:* return survivor to informed PCP/help survivors identify knowledgeable providers; *PCP: accept survivors into practice and learn about survivorship needs*<br>Connect survivors with survivorship resources and services<br>Conduct guideline-concordant surveillance for long-term and late effects<br>Manage symptoms/late effects<br>Educate survivors about late effects<br>Assess psychosocial needs and provide psychosocial support<br>Counsel regarding healthy behaviors<br>Coordinate care<br>Refer to appropriate specialists (medical, legal, financial)
Survivor health behaviors	Receive surveillance for long-term and late effects and appropriate preventive care<br>Receive social support, nutritional, rehabilitative, and fertility preservation services<br>Health behaviors<br>Alcohol/Tobacco/Other drugs<br>Physical activity<br>Loss to follow-up<br>Emergency department visits<br>Hospitalizations
Health status	Mortality<br>Morbidity/Late effects<br>Peripheral neuropathy<br>Cardiac dysfunction<br>Cognitive problems<br>Bone loss<br>Infertility<br>Sexual dysfunction<br>Insomnia<br>Obesity<br>Quality of life (health-related)/Functional status<br>Fatigue<br>Pain<br>Vasomotor and menopausal symptoms<br>Psychosocial needs<br>Depression<br>Anxiety
Consumer satisfaction	
Costs	

[a]Edits made between Initial Program Theory and Refined Program Theory are *italicized*. PCP = primary care provider

Supplementary Figure 1. Steps from development of the Initial Program Theory to Refined Program Theory

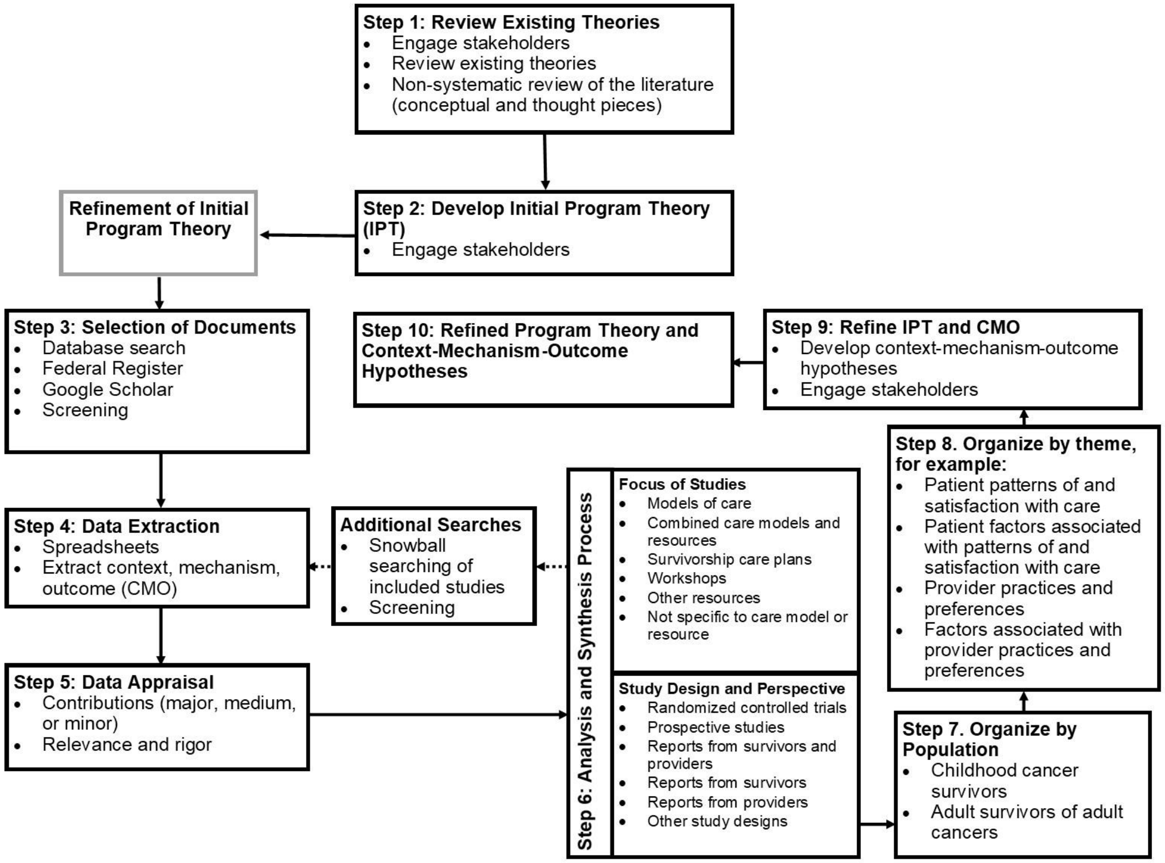

Supplementary Figure 2. Literature search results

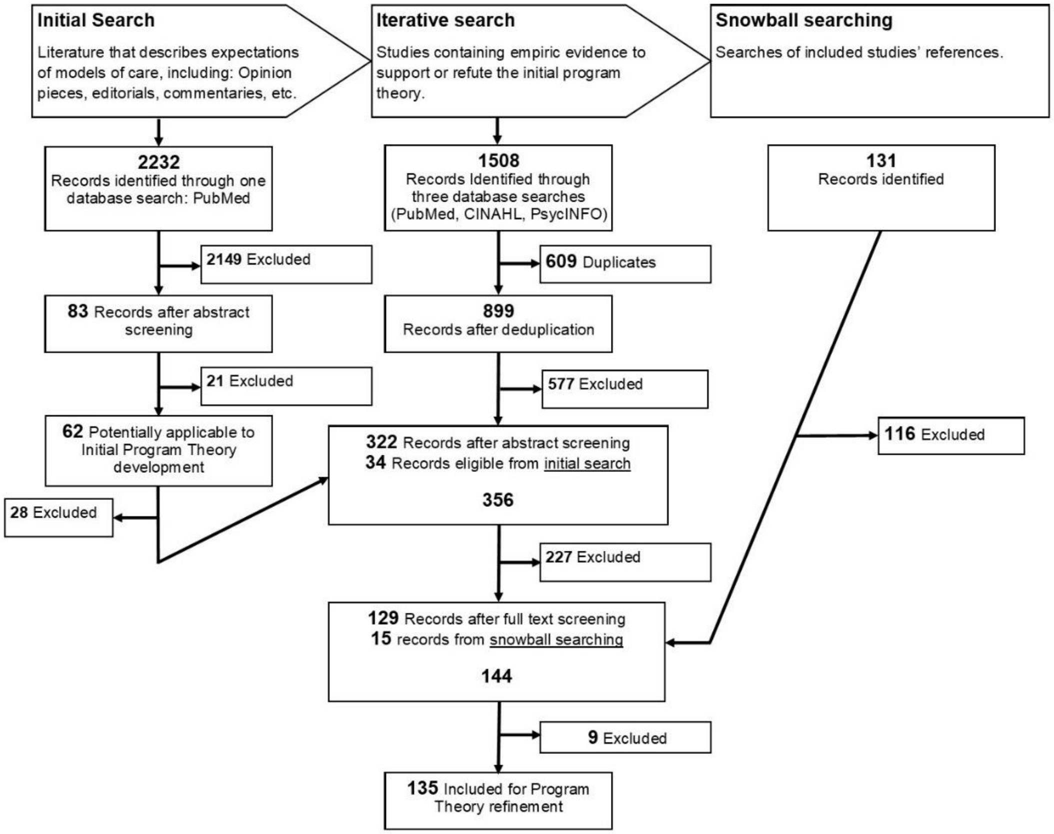

Supplement: pkac012_Supplementary_Data [file pkac012_supplementary_data.pdf]
